# Local hypoxia is produced at sites of intratumour injection

**DOI:** 10.1038/sj.bjc.6600059

**Published:** 2002-02-01

**Authors:** P L Olive, C-M Luo, J P Banáth

**Affiliations:** Medical Biophysics Department, British Columbia Cancer Research Centre, 601W 10th Avenue, Vancouver, BC, V5Z 1L3 Canada

**Keywords:** tumour hypoxia, pimonidazole, intratumour injection

## Abstract

Intratumour injection, commonly used for gene or drug delivery but also associated with needle biopsy or insertion of invasive measuring devices, may damage tumour microvessels. To examine this possibility, SCCVII tumours grown subcutaneously in C3H mice were injected with a 26 gauge needle containing 0.1 ml of the fluorescent dye Hoechst 33342 to label cells lining the track of the needle. Hoechst-labelled cells sorted from these tumours were more sensitive to killing by hypoxic cell cytotoxins (tirapazamine, RSU-1069) and less sensitive to damage by ionizing radiation. Hoechst-labelled cells also bound the hypoxia marker pimonidazole when given by i.p. injection. Intratumour injection transiently increased hypoxia from 18 to 70% in the tumour cells adjacent to the track of the needle. The half-time for return to pre-treatment oxygenation was about 30 min; oxygenation of tumour cells along the track had recovered by 20 h after intratumour injection. This effect could have significant implications for intratumour injection of drugs, cytokines or vectors that are affected by the oxygenation status of the tumour cells as well as potential effects on biodistribution via local microvasculature.

*British Journal of Cancer* (2002) **86**, 429–435. DOI: 10.1038/sj/bjc/6600059 www.bjcancer.com

© 2002 The Cancer Research Campaign

## 

Intratumour injection is used to deliver a variety of agents, including drugs, genes and cytokines that are available in limited amounts or that do not normally localize to tumours (Graeber *et al*, 1999; Nemunaitis *et al*, 1999). Delivering chemotherapy drugs directly to tumours should produce a higher drug concentration in the tumour than in neighbouring normal tissues (Amano *et al*, 1988). However, one of the concerns of intratumour injection is that inhomogeneities in drug delivery within the tumour may result in failure to adequately expose all of the tumour cells to the agent (McDouall and Evelhoch, 1990).

Therefore, experiments (reported here) were designed initially to measure the distribution of drugs given by intratumour injection. The bioreductive hypoxic cell cytotoxin tirapazamine was injected together with a rapidly bound fluorescent dye, Hoechst 33342, directly into SCCVII murine tumours. Hoechst 33342 binds to cells lining the track of the needle, but tirapazamine was expected to diffuse to hypoxic regions within the tumours. Interestingly, tumour cells most sensitive to tirapazamine were those cells that bound the most Hoechst 33342. This led us to question whether intratumour injection, and presumably other invasive procedures, may affect local tumour oxygenation.

Our previous efforts to demonstrate an effect of intratumour needle puncture on average tumour oxygenation in experimental tumours were inconclusive (Aquino-Parsons *et al*, 1999). Although heterogeneity in hypoxic fraction was increased in tumours that underwent puncture with an Eppendorf oxygen electrode (tip dimension 250 microns), there was no significant change in median tumour oxygenation 24 h after tumour puncture when measured using the alkaline comet assay to detect hypoxic cells. However, the inability to detect significant changes in oxygenation was complicated by intertumour variability in oxygenation and by the possibility that only those cells adjacent to the needle track might be affected by the procedure.

To examine the hypoxic status of cells along the needle track, we used the fluorescent dye Hoechst 33342 in combination with the hypoxic cell marker pimonidazole to measure the duration and extent of hypoxia. The importance of Hoechst drug dose and injection volume were also examined as possible contributors to hypoxia.

## MATERIALS AND METHODS

### SCCVII tumours and bioreductive drugs

All procedures were approved by the Animal Care Committee at the University of British Columbia, and mice were maintained in a holding facility approved by the Canadian Council on Animal Care. All procedures conform in every way to the UKCCR Guidelines (UKCCR, 1998). SCCVII squamous cell carcinoma cells (Suit *et al*, 1985), originally obtained from Dr M Horsman, were transplanted subcutaneously over the sacral region of inbred male C3H/HeN mice, approximately 30 gm in weight. Tumours were used for experimentation 2–4 weeks later when they had reached a weight of approximately 400–500 mg. Tirapazamine and RSU-1069 were kindly provided by Sanofi-Winthrop and by Parke-Davis Pharmaceuticals respectively. Tirapazamine was dissolved in phosphate buffered saline (PBS) and RSU-1069 was dissolved in PBS, pH 4, immediately before use.

### Pimonidazole binding to identify hypoxic cells

To detect hypoxic cells in tumours, mice were injected intraperitoneally with 100 mg kg^−1^ pimonidazole hydrochloride (Hypoxyprobe-1, NPI Inc., Belmont, MA, USA) from a stock solution containing 20 mg ml^−1^ in sterile PBS. In one experiment, pimonidazole was injected i.t. at a dose of 2–50 mg kg^−1^. Ninety minutes after intraperitoneal injection of pimonidazole, corresponding to three plasma half-lives for this drug (Walton *et al*, 1989), mice were sacrificed. Tumours were excised and a single cell suspension was prepared by mincing in ice-cold PBS and incubating for 30 min at 37°C with a mixture of trypsin, collagenase and DNase as described previously (Olive, 1989). Cells were then filtered through 30 μm nylon mesh, centrifuged and resuspended in cold PBS. Cells were fixed in 70% ethanol and refrigerated overnight or up to several days before analysis of pimonidazole binding.

### Intratumour injection of Hoechst 33342 and cell sorting

SCCVII tumours were injected using a 26 gauge needle passed through the overlying skin into the tumour. The angle of injection was changed to create three tracks through the tumour using the same entry site, and typically 0.1 ml Hoechst 33342 dissolved in PBS was slowly injected. Single cells were prepared using the enzyme disaggregation method, and cells were incubated with fluorescein-isothiocyanate conjugated anti-mouse IgG to discriminate tumour cells from macrophages (Olive, 1989). Cells were sorted on the basis of the Hoechst concentration gradient, as previously described. Sorted cells were analyzed for pimonidazole binding, or for cell viability using a standard clonogenicity assay. The plating efficiency of cells from untreated SCCVII tumours was 0.3–0.4.

### Flow cytometry analysis of pimonidazole binding

Ethanol-fixed cells were centrifuged, washed once in PBS then resuspended in PST (PBS containing 4% serum and 0.1% Triton X-100). A FITC-conjugated (1 : 1000 dilution) primary antibody was added to 2×10^6^ alcohol-fixed cells for 2 h at 37°C (for antibody source and description, see Raleigh *et al*, 1999). Samples were rinsed in PST and those cells that were incubated with the unconjugated primary were resuspended in a FITC conjugated secondary antibody diluted 1 : 100 in PST for 1 h at 37°C. Samples were rinsed in PST and resuspended for DNA staining in 1 ml PBS containing 1 μg ml^−1^ 4,6-diamidino-2-phenylindole dihydrochloride hydrate. Samples were analyzed on a Coulter Epics Elite cell sorter (Beckman Coulter Corp. Hialeah, FL, USA).

Univariate histograms, plotted as cell number *vs* logarithm of fluorescence anti-pimonidazole antibody intensity, were analyzed by a least-squares approach for three normal curves representing aerobic, intermediate and hypoxic tumour cell populations. The hypoxic fraction was defined as the percentage of the population that bound, on average, 10 times more pimonidazole than the aerobic population of cells (Olive *et al*, 2000). Since the SCCVII tumour is tetraploid, any normal cells with diploid DNA content were easily eliminated from analysis.

### Image analysis of pimonidazole binding

To examine co-localization of Hoechst 33342 and pimonidazole in frozen sections, SCCVII tumours were embedded in OCT compound and frozen sections (approximately 7 micron diameter) were prepared using a cryostat. Sections were examined immediately with UV excitation using a Zeiss axioplan epi-fluorescence microscope with CCD camera and image software. Areas that showed significant Hoechst 33342 fluorescence (especially longitudinal sections of blood vessels) were captured. Then sections were fixed in 70% ethanol (Hoechst 33342 is lost at this step) and stained for pimonidazole using the FITC-conjugated anti-pimonidazole monoclonal antibody (1 : 1000 dilution in PST). The same area of the tumour was re-examined using 488 nm excitation. Digitized images were pseudo-coloured and superimposed using Picture Publisher software.

### Analysis of DNA damage and hypoxic fraction using the comet assay

DNA single-strand breaks produced by tirapazamine were measured using the alkaline comet assay as previously described (Olive, 1995). DNA damage was quantified as an increase in tail moment, an indicator of damage that is proportional to the number of strand breaks per cell (Olive *et al*, 1990). Tail moment is defined as the product of the per cent of DNA (fluorescence) in the tail and the distance between the means of the head and tail fluorescence distributions.

## RESULTS

Figure 1

**Figure 1:**
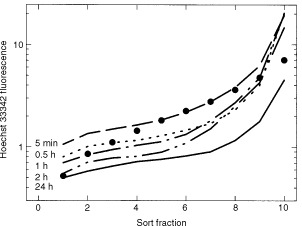
SCCVII tumours (400–500 mg) were injected with 20 ng Hoechst 33342 in 0.1 ml along three tracks within the tumour. Tumours were removed at the times indicated, and a single cell suspension was prepared for analysis using a Becton-Dickinson FACS 440 cell sorter. The solid circles indicate the distribution of 1600 ng Hoechst 33342 given by a single i.v. injection.

shows the distribution of Hoechst 33342 when injected into SCCVII tumours via a 26 gauge needle (400 micron diameter) inserted through the skin overlying the subcutaneous tumour with three slow passes through the tumour from the same entry site. A total of 0.1 ml Hoechst 33342 (20 ng) was delivered. When tumours were injected with 0.1 ml PBS or with 0.1 ml PBS containing 20 ng Hoechst 33342, the overall hypoxic fraction was not significantly different (18.6±12.1 *vs* 20.8±7.4, mean and standard deviation, *n*=6). Five minutes to 24 h later, tumours were excised, and a single cell suspension was prepared. Single cells were then analyzed by flow cytometry. Fluorescence within the fractions from an individual tumour varied 20–40-fold when tumour cells were assigned to 10 equal fractions. Fluorescence intensity increased dramatically in fractions 9 and 10, representing 10–20% of the tumour cells.

Intensity decreased slightly over the first 2 h following intratumour injection but was 4–5 times lower by 24 h after injection. Intravenous injection of 80 times more Hoechst 33342 (1.6 mg per mouse) produced a similar average intensity but a reduced maximum intensity (black circles) consistent with a larger percentage of tumour cells being exposed to the dye when delivered by intravenous injection.

**Figure 2:**
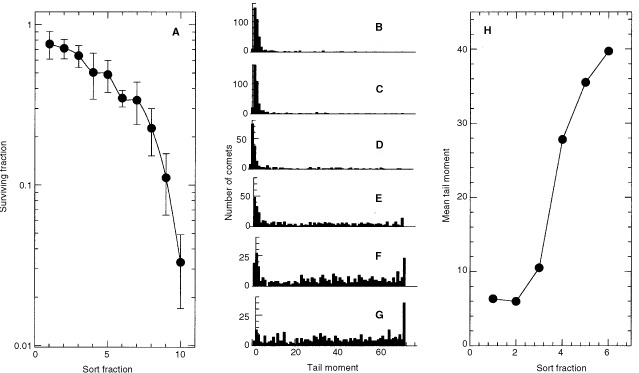
SCCVII tumours were injected with 0.1 ml PBS containing 0.1 mg tirapazamine and 0.1 mg Hoechst 33342. Sixty minutes later, tumours were excised and a single cell suspension was prepared. Cell sorting was used to select cells on the basis of Hoechst 33342 fluorescence so that fraction 10 out of 10 in (**A**) or six out of six in (**H**) represent the most fluorescent fraction of cells within the tumour (i.e., the cells closest to the needle track). (**A**) Shows the mean and standard deviation for clonogenic survival for three tumours injected with 0.1 mg tirapazamine. (**H**) Shows the average tail moment for (**B**–**G**) that indicate the wide heterogeneity in DNA damage within individual cells of the six sort fractions.

The distribution of DNA damage within SCCVII tumours after intratumour injection of tirapazamine together with Hoechst 33342 is shown in Figure 2

. Cells were selected by fluorescence activated cell sorting based on the gradient of Hoechst 33342 fluorescence intensity, with the expectation that the brightly fluorescent cells would be those cells closest to the site of injection. Sorted populations were subsequently analyzed for DNA damage using the alkaline comet assay, and for cell survival using a clonogenicity endpoint. Cells that contained the most Hoechst 33342 also showed the most killing and DNA damage by tirapazamine. Similar results were obtained using another bioreductive drug that also preferentially kills hypoxic cells, RSU-1069 (data not shown).

**Figure 3:**
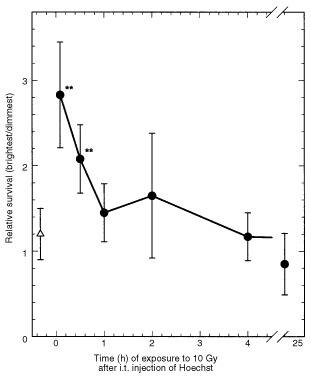
Effect of ionizing radiation on survival of cells lining the needle track. Tumours were injected with 20 ng Hoechst 33342 in 0.1 ml. Five minutes to 20 h later, tumours were exposed to 10 Gy X-rays and subsequently excised. Single cells were sorted based on Hoechst 33342 concentration into six fractions and plated for colony formation. The relative clonogenic survival of the brightest cells compared to the dimmest cells of the tumour is plotted as a function of time of irradiation after Hoechst injection. The mean and standard deviation for five tumours is shown. Note that when Hoechst 33342 is given by i.t. injection after irradiation (▵), there is no distinction between the sensitivity of the dimmest and brightest cells. ** Indicate values significantly different from the response when irradiation is given post-injection.

Since the drug concentration was highest in cells along the track of the needle, one could argue that more damage occurred here simply because these cells were exposed to the highest drug dose. Therefore, response to ionizing radiation, which produces three times more killing in aerobic than hypoxic cells, was also used to determine whether cells bordering the needle track were hypoxic. Tumours that had been injected with Hoechst 5 min before exposure to radiation showed less killing of the brightly fluorescent cells compared to the dimmest cells (Figure 3

), supporting the idea that cells next to the site of injection were hypoxic. This protective effect decreased with time after injection, indicating either a return of oxygen to these cells or perhaps their loss. As expected, the average response of the population was not significantly different than the response of the dimmest population.

**Figure 4:**
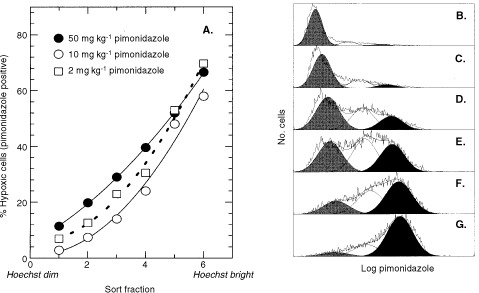
SCCVII tumours were injected in three tracks with 0.1 ml PBS containing 0.1 mg Hoechst 33342 and either 2, 10 or 50 mg kg^−1^ pimonidazole. Tumours were removed 90 min later. Although the average antibody intensity within the tumours increased six-fold over this dose range, the percentage of cells that bound 10 times more pimonidazole than the ‘aerobic’ population of cells was similar. (**A**) Shows the percentage of hypoxic cells for each sort fraction. (**B**–**G**) Show flow histograms and curve fitting for pimonidazole antibody-stained cells sorted from the 2 mg kg^−1^ tumour. The distribution for the hypoxic cells (indicated in black) is shown for the six sort fractions from the dimmest Hoechst-stained cells (**B**) to the brightest Hoechst-stained cells (**G**).

To directly confirm that cells lining the needle track after intratumour injection were hypoxic, the hypoxia marker pimonidazole was administered simultaneously with the Hoechst dye. Tumour cells were also sorted on the basis of the Hoechst 33342 concentration gradient, and sorted cells were fixed and subsequently analyzed for pimonidazole binding using anti-pimonidazole antibodies. Regardless of the concentration of pimonidazole injected into the tumour, the brightly fluorescent cells showed 60–70% hypoxic cells while the dimly fluorescent cells contained only 3–11% hypoxic cells (Figure 4

).

**Figure 5:**
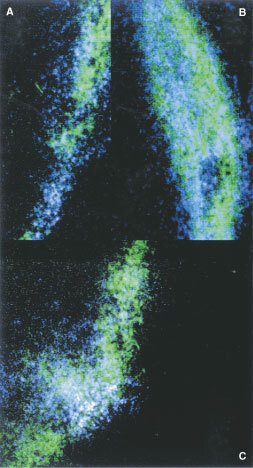
Three digitized images of frozen sections of SCCVII tumours injected i.t. with Hoechst 33342 5 min after i.p. injection of 100 mg kg^−1^ pimonidazole. The blue-stained nuclei indicate the regions that stained with Hoechst 33342. The green stained cells bound FITC-conjugated anti-pimonidazole antibodies. The remaining black background shows unstained regions of the tumours.

Delivering pimonidazole i.p. at various times before and after intratumour injection of Hoechst 33342 provided more convincing evidence that cells lining the needle track were hypoxic. Figure 5

shows three digitized images of frozen sections of tumours given an i.p. injection of pimonidazole followed 5 min later by i.t. injection of Hoechst 33342. Regions of tumours labelled with Hoechst 33342 were identified and imaged. Then after fixation and incubation with anti-pimonidazole antibody, the same regions were imaged again. Superimposed images indicate clear co-localization of pimonidazole and Hoechst 33342.

**Figure 6:**
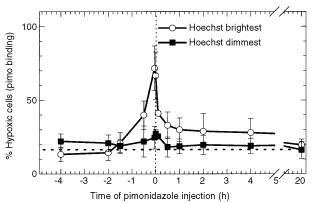
Relation between time of intratumour injection and increase in binding of pimonidazole in SCCVII tumours. Mice were given i.t. injections with Hoechst 33342 at time ‘0’. Pimonidazole (100 mg kg^−1^) was injected i.p. 4 h before to 20 h after i.t. injection of Hoechst. Ninety minutes after pimonidazole injection, tumours were excised and single cells were sorted into six fractions on the basis of Hoechst concentration. Sorted samples were analyzed for the percentage of hypoxic cells in the Hoechst dimmest one out of six and brightest one out of six of the tumour cells. The means and standard deviations for 3–6 tumours per time point are shown.

To quantify this effect, pimonidazole was administered i.p. at various times before and after i.t. injection of Hoechst 33342. Single cells from these tumours were sorted on the basis of Hoechst 33342 concentration into populations representing the brightest one out of six of the tumour cells and the dimmest one out of six of the cells. These sorted populations were then analyzed for pimonidazole adducts. When pimonidazole was injected 90 min or more before intratumour injection of Hoechst, there was no significant difference between the hypoxic fraction of the Hoechst brightest or dimmest cells, that is, the hypoxic fraction was similar for cells lining the track of the needle or distant from the track (Figure 6

). This result is expected since the plasma half-life of pimonidazole in mice is only about 30 min so most of the pimonidazole will have already bound to existing hypoxic cells before the Hoechst was administered. When pimonidazole was given i.p. 30 min or less before Hoechst 33342 injection, then there was sufficient plasma pimonidazole to bind to any regions of hypoxia created by the intratumour injection. In fact, when administered within 5 min of each other, the hypoxic fraction of the Hoechst brightest cells increased to almost 70%. For at least 30 min following intratumour injection, the hypoxic fraction remained significantly elevated. Pooled values from 1 to 4 h were significantly elevated in the brightest cells compared to the dimmest population when analyzed using a paired *t*-test. By 20 h after intratumour injection, hypoxic fraction in the cells lining the needle track returned to normal levels (Figure 6).

**Figure 7:**
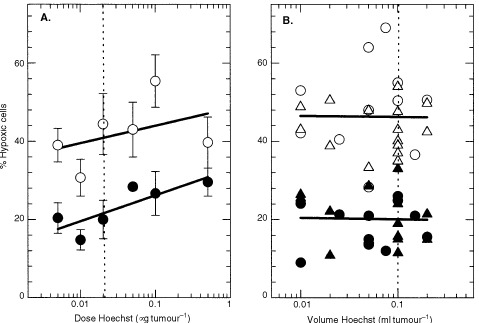
Effect of Hoechst 33342 injection dose and injection volume on hypoxic fraction measured using pimonidazole binding. (**A**) SCCVII tumours were injected in three tracks with 0.1 ml containing various amounts of Hoechst 33342 administered 5 min after i.p. injection of 100 mg kg^−1^ pimonidazole. The means and standard deviations for 3–5 tumours per dose are shown. (**B**) SCCVII tumours were injected in three tracks with either the same amount of Hoechst (20 ng, triangles) or the same concentration of Hoechst (1 mg ml^−1^, circles) in different volumes of PBS given 5 min after i.p. injection of pimonidazole. Results for individual tumours are shown. The dotted lines indicate the dose and volume used in Figures 3 and 6. Open symbols show the response of the brightest one out of six of the tumour cells and closed symbols show the response of the dimmest one out of six of the cells.

Hoechst 33342 is known to be vasoactive at high concentrations (Trotter *et al*, 1990), so intratumour injection of this drug could lead to a localized decrease in blood flow and might explain the hypoxia associated with the injection site. Alternatively, injection of a significant volume of fluid into a tumour could increase interstitial fluid pressure, leading to vascular collapse and a subsequent increase in hypoxia. To examine these possibilities, different concentrations and volumes of Hoechst 33342 were injected 5 min after i.p. administration of pimonidazole. Although there was a trend for the hypoxic fraction to increase with increasing Hoechst 33342 concentration (Figure 7a

), it is unlikely that Hoechst alone was responsible for the effect observed in Figure 6. A 20-fold variation in fluid volume injected had no apparent effect on the measured hypoxic fraction when the same amount (20 ng) or concentration (1 mg ml^−1^) of Hoechst was injected (Figure 7b).

## DISCUSSION

Intratumour injection is widely used to administer genes, drugs and cytokines. In this situation, small gauge needles are inserted along with some volume of fluid, generally in several passes through the tumour. We found that intratumour injection of the hypoxic cell specific cytotoxin tirapazamine produced cell killing and DNA damage in SCCVII tumour cells closest to the needle track in spite of our expectation that this drug should be able to diffuse to hypoxic areas of the tumour (Figure 2). This led us to examine whether local regions of hypoxia might be produced by intratumour injection. Results using ionizing radiation and pimonidazole as methods to detect hypoxic cells confirmed that cells close to sites of intratumour injection were significantly more hypoxic than cells distant from the injection sites.

The effects we have observed here are confined to the immediate region of cells surrounding the sites of injection. This observation is consistent with previous studies that found only a small region of the RIF-1 tumour surrounding the site of D_2_O injection was actually labelled with this tracer as measured using NMR imaging (McDouall and Evelhoch, 1990). In these studies, RIF-1 tumours were injected with 0.1 ml D_2_O and the label could not be detected after about 10 min. Higher volumes (0.4 ml) labelled a larger area of the tumour but were cleared at the same rate. This suggests that the slower recovery kinetics observed in this study is more likely to represent repair of damage to microvessels rather than clearance of the injected fluid. Jain and colleagues also noted that using a very slow infusion rate from a single point source, bulk flow was responsible for removal of fluid and reabsorption via blood vessels was minimal in the LS174T xenograft tumour (Boucher *et al*, 1998). Although the authors suggested that stagnant blood flow in the center of these tumours might be responsible, this result could also be explained if blood vessels were damaged in the region surrounding the infusion needle.

The typical hypoxic fraction in the SCCVII tumour of air-breathing mice is about 12% when measured using pimonidazole binding (Olive *et al*, 2000). However, 5 min following intratumour injection, this increased to 18–20%. This is consistent with expected results if 10% of the cells of the tumour are made hypoxic by the passage of the needle three times through this tumour volume. Since we were using a potentially vasoactive dye, Hoechst 33342, to delineate the cells bordering the needle track, we could not rigorously exclude the possibility that this fluorescent drug was responsible for producing vasoconstriction and hypoxia where the concentration was greatest. However, we did not observe significant changes in hypoxic fraction along the track when the Hoechst concentration was varied over 10-fold. The time course studies shown in Figures 3 and 6 indicate that the local hypoxia produced by intratumour injection is maximum within the first few minutes after injection and that recovery occurred quite rapidly over the first 30 min then continued gradually over the next several hours. Although loss of Hoechst 33342-labelled cells might explain this recovery, Hoechst 33342 intensity in the brightest populations remained high for the first few hours after intratumour injection (Figure 1), arguing against significant cell loss.

Localized damage to the tumour blood vessels caused by invasive procedures is not unexpected. Previous reports have indicated that invasive techniques, such as fine needle aspiration biopsy, can occasionally produce extensive tissue damage (e.g. Gordon *et al*, 1993; Lee *et al*, 1994). Insertion of needle electrodes is also part of invasive procedures including measurements of pH or oxygen, measurement of interstitial fluid pressure and laser Doppler measurements of tumour blood flow. These procedures differ from intratumour injections in that no fluid is administered. Moreover, the Eppendorf oxygen electrode continuously advances through the tissue making it unlikely that any damage by the electrode will contribute to the measurements. Needle size is also likely to be important, and the tip of the Eppendorf oxygen electrode tapers to about 250 microns (compared to 400 microns for a 26 gauge needle). Also, as the tip of the oxygen electrode is relatively blunt compared to a needle, there should be less damage to the microvessels. The question of oxygen electrode damage to tumours has been addressed previously (Steinberg *et al*, 1995). These authors examined tissue destruction along the needle tracks, noting microvessel rupture and destroyed sinusoids. They concluded that local oxygen tension measurements should be completed quickly and performed only in previously undamaged regions of the tumour. Microelectrode damage was also found to be largely confined to the tissue track at puncture sites in arteries (Cole *et al*, 1983). Previous results from our laboratory examined the effect of passing an Eppendorf electrode four times through the SCCVII tumour on hypoxic fraction measured using the comet assay (Aquino-Parsons *et al*, 1999). No significant change in average tumour oxygenation was observed 5 min after tumour puncture. In the same study, the calculated hypoxic fraction from clonogenic measurements was also found to be the same for control tumours and tumours that were punctured with an Eppendorf electrode 5 min before irradiation. These results are consistent with the idea that immediate changes in tumour oxygenation are confined largely to the region of cells adjacent to the needle track. Since only a few per cent of the tumour cells are affected, this difference is unlikely to be detected in heterogeneous animal tumour models when only average responses are measured.

The fact that adjacent tumour cells are hypoxic and gradually reoxygenate has implications for the use of intratumour injection to deliver drugs. Biodistribution of agents delivered by this route is likely to be affected by damage to tumour microvessels. In addition, oxygen dependency for toxicity has been demonstrated for ionizing radiation, cytokines and several anticancer drugs (for a review, see Hockel and Vaupel, 2001). The hypoxic microenvironment could explain why photodynamic therapy was not improved by intratumour injection of haematoporphyrin in spite of increasing the drug concentration compared to i.p. administration (Lin *et al*, 1988). Toxicity of TNF-α is also known to be highly dependent on tumour cell oxygenation status (Lynch *et al*, 1995). Hypoxia- or stress-induced upregulation of genes such as cyclooxygenase-2, TP53, and inducible nitric oxide synthase, may also influence the effect of agents injected directly into tumours.
